# Prognostic model integrating the blood urea nitrogen-to-serum albumin ratio and radiological features for poor functional outcome in cerebral venous sinus thrombosis

**DOI:** 10.3389/fneur.2026.1953972

**Published:** 2026-10-21

**Authors:** Jiawei Zhao, Kai Liu, Shen Li, Hongbing Liu, Yuming Xu, Zongping Xia, Bo Song

**Affiliations:** 1 Department of Neurology, The First Affiliated Hospital of Zhengzhou University, Zhengzhou, Henan, China; 2 NHC Key Laboratory of Prevention and Treatment of Cerebrovascular Diseases, Zhengzhou, Henan, China; 3 Clinical Systems Biology Laboratories, The First Affiliated Hospital of Zhengzhou University, Zhengzhou, Henan, China

**Keywords:** blood urea nitrogen to serum albumin ratio, cerebral venous sinus thrombosis, nomogram, outcomes, straight sinus thrombosis

## Abstract

**Background:**

The blood urea nitrogen-to-serum albumin ratio (BAR) is a novel composite biomarker. This study investigated whether BAR could reliably predict poor functional outcomes in patients with cerebral venous sinus thrombosis (CVST).

**Methods:**

All patients with CVST enrolled in this observational study between January 2011 and December 2025 were identified. The study population was randomly divided into a derivation cohort comprising 70% of the patients and a validation cohort comprising the remaining 30%. Logistic regression analysis was performed to identify independent predictors of poor functional outcomes. A prognostic nomogram was developed using data from the derivation cohort, and its predictive performance was assessed in the validation cohort.

**Results:**

A total of 803 patients were included in this study. In the derivation cohort, coma, older age, intracerebral hemorrhage, straight sinus thrombosis, and higher BAR were independently associated with poor functional outcomes and were incorporated into the nomogram. Straight sinus thrombosis was independently associated with higher odds of poor functional outcome (odds ratio [OR], 4.525; 95% confidence interval [CI], 2.267–9.031), whereas each one-unit increase in BAR was associated with an OR of 1.411 (95% CI, 1.226–1.625). In the derivation cohort, the area under the receiver operating characteristic curve was 0.900, compared with 0.893 in the validation cohort.

**Conclusion:**

Higher admission BAR was independently associated with poor functional outcomes in patients with CVST. The nomogram incorporating BAR and radiologically confirmed straight sinus thrombosis may facilitate early individualized risk stratification.

## 1 Introduction

As an infrequent cerebrovascular disorder, cerebral venous sinus thrombosis (CVST) accounts for approximately 0.5–1% of all cases of stroke (1–3). Thrombotic occlusion of the cerebral veins or dural venous sinuses impairs cerebral venous outflow, resulting in increased venous pressure, cerebral edema, intracranial hemorrhage, and venous infarction (4, 5). In contrast to arterial stroke, CVST disproportionately affects younger adults, particularly women of reproductive age, with oral contraceptive use, pregnancy, and the puerperium representing well-recognized predisposing factors (6). Its clinical presentation is highly heterogeneous, ranging from isolated or subacute symptoms of intracranial hypertension, such as headache, nausea, papilledema, and visual disturbances, to focal neurological deficits, cranial neuropathies, seizures, encephalopathy, and coma (7–11). Symptoms of venous stroke usually develop more gradually than those of arterial stroke, although some patients may experience an acute or fulminant course that can hamper timely diagnosis and early risk assessment. The diagnosis of CVST depends primarily on neurovascular imaging. The two main modalities for diagnostic confirmation are computed tomography venography and magnetic resonance venography (2, 12, 13). Nonetheless, although imaging is essential for determining the site and extent of venous thrombosis and identifying associated parenchymal lesions, radiological findings alone are insufficient to characterize the systemic physiological and metabolic status of individual patients. By integrating imaging features with readily obtainable clinical and laboratory biomarkers, prognostic evaluation and early risk stratification may be improved (14).

Blood urea nitrogen (BUN) is a routine biochemical measure of the concentration of circulating urea nitrogen, which is produced from protein catabolism in the liver and excreted mainly by the kidneys (15). In addition to its use in assessing renal function, BUN has emerged as an important prognostic marker for cardiovascular and cerebrovascular diseases because it may reflect changes in intravascular volume, renal perfusion, and systemic catabolic stress (16–19). Serum albumin (ALB) levels are influenced by nutritional status, systemic inflammation, vascular permeability, and overall disease severity. Low ALB levels are often associated with poor functional outcomes in vascular diseases (20–22). The blood urea nitrogen-to-albumin ratio (BAR) is a readily available composite biomarker that can be easily assessed in clinical practice and may reflect metabolic, inflammatory, and nutritional disturbances (23, 24). Accumulating evidence indicates that an elevated BAR predicts mortality and poor functional outcomes in several acute cardiovascular and cerebrovascular conditions (25–27).

Nonetheless, although BAR has been shown to have prognostic value in various cerebrovascular diseases, its association with clinical outcomes in CVST remains unclear. Therefore, we aimed to assess the prognostic value of BAR in patients with CVST in conjunction with radiological findings. We also evaluated whether combining BAR with relevant clinical and radiological characteristics could improve the prediction of poor functional outcomes and support personalized prognostic stratification.

## 2 Materials and methods

### 2.1 Study design and study population

This exploratory analysis used data from the Henan CVST Registry maintained at the First Affiliated Hospital of Zhengzhou University. A total of 1,108 patients with CVST were screened between January 2011 and December 2025 (Figure 1). The diagnosis was established according to consensus guidelines, and standardized radiological assessments were performed to confirm venous sinus occlusion (Figure 2). Baseline imaging characteristics, including ischemic infarction and intracranial hemorrhage, were evaluated using the initial brain computed tomography and/or magnetic resonance imaging examinations performed at admission. A period of ≤30 days from symptom onset to admission defined the acute or subacute phase (28).

**Figure 1:**
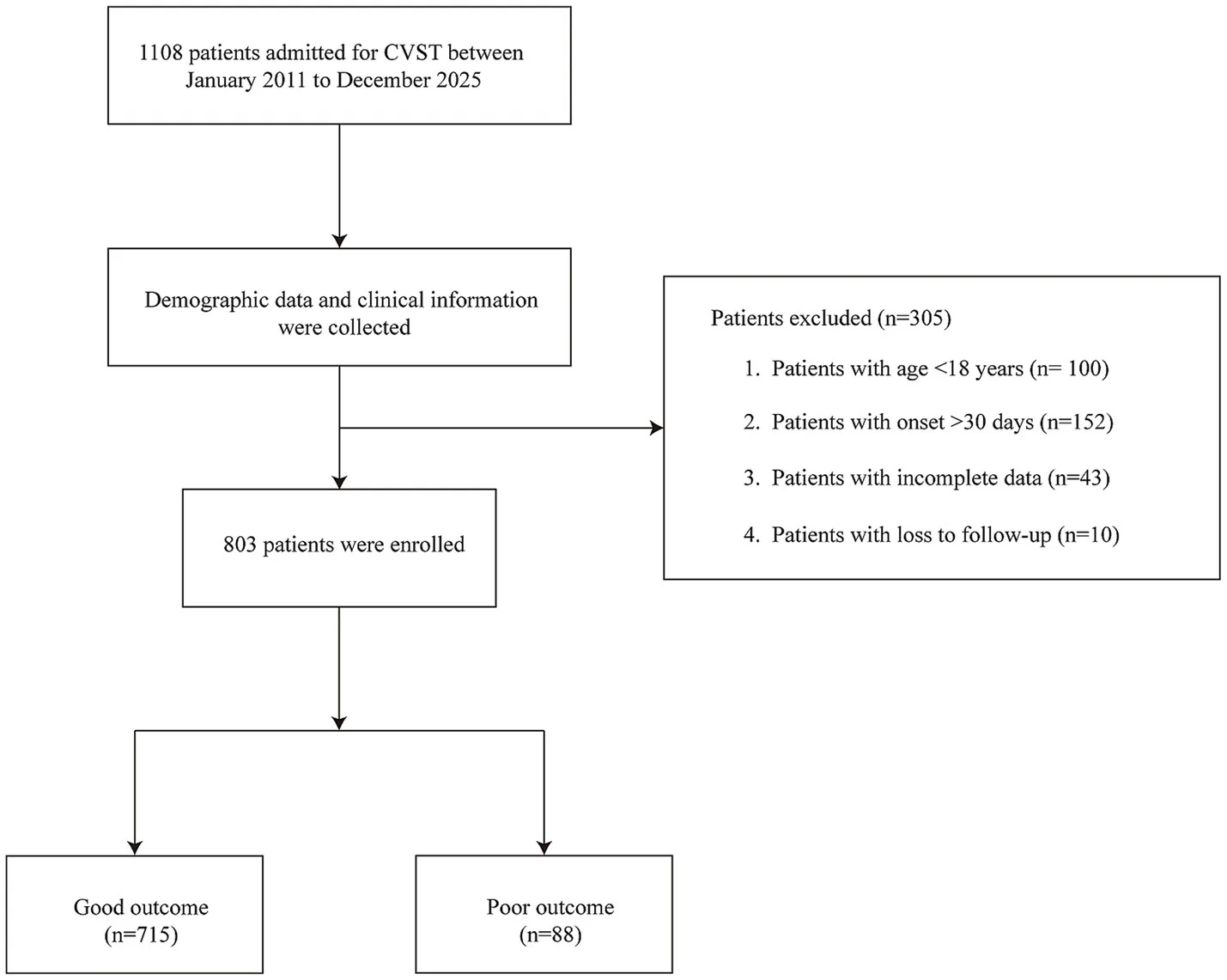
Flowchart illustrating patient selection and the study design.

**Figure 2:**
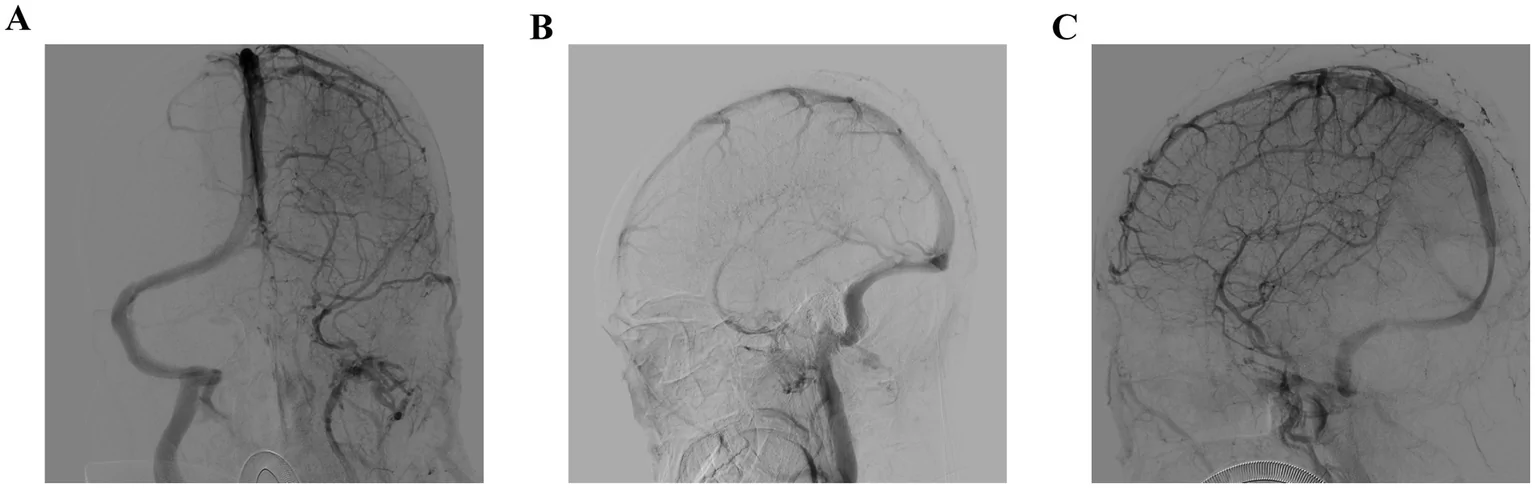
Representative digital subtraction angiography (DSA) images showing cerebral venous sinus thrombosis at different anatomical locations. **(A)** Thrombosis involving the transverse and sigmoid sinuses. **(B)** Thrombosis involving the superior sagittal sinus. **(C)** Thrombosis involving the straight sinus.

Patients were excluded according to the following prespecified criteria: (1) age <18 years; (2) symptom duration >30 days before hospitalization; (3) missing baseline BUN or ALB data; and (4) missing follow-up data. The study was approved by the Ethics Committee of the First Affiliated Hospital of Zhengzhou University (approval number: 2024-KY-0838-001) and was conducted in accordance with the Declaration of Helsinki.

### 2.2 Data collection

Blood samples were obtained from patients within 24 h of hospitalization to establish baseline BUN and ALB levels. Demographic, clinical, and radiological data were extracted from the registry, including age, sex, presenting symptoms, vascular risk factors, and baseline neuroimaging findings.

### 2.3 Definitions

BAR was calculated by dividing the baseline BUN concentration (mg/dL) by the corresponding ALB concentration (g/dL) (29).

### 2.4 Outcome assessment

Functional outcomes were assessed at 6 months after the index hospitalization using the modified Rankin Scale (mRS). A poor functional outcome was defined as an mRS score of 3–6, whereas a good functional outcome was defined as an mRS score of 0–2 (30). Follow-up assessments were conducted through telephone interviews by trained neurologists with expertise in cerebrovascular diseases. The neurologists responsible for follow-up evaluation were blinded to the baseline BAR values and prognostic model results.

### 2.5 Statistical analysis

The distribution of the data determined how continuous variables were presented. Continuous variables that met the normality assumption were reported as the mean ± standard deviation, whereas variables with skewed distributions were summarized as the median and interquartile range. Continuous variables were compared using the Student’s *t*-test or Mann–Whitney *U* test, as appropriate. Categorical variables were summarized as frequencies and percentages, and between-group differences were assessed using Pearson’s chi-square test or Fisher’s exact test, as appropriate. To assess model generalizability, the cohort was randomly divided into derivation and validation cohorts at an approximate ratio of 7:3. The model was developed in the derivation cohort and subsequently evaluated in the validation cohort. To further assess the robustness of predictor selection and model performance, additional sensitivity analyses were performed using the full cohort. In addition, internal validation was performed using 10-fold cross-validation and bootstrap resampling with 1,000 repetitions. The threshold for statistical significance was set at a two-sided *p* < 0.05.

The modeling workflow for developing and validating the prognostic nomogram consisted of several steps. Variables were first assessed for their univariate associations with poor functional outcomes in patients with CVST using the derivation cohort. Variables meeting the prespecified significance threshold (*p* < 0.05) were subsequently entered into a multivariable logistic regression model to identify independent predictors of outcome. The final nomogram was constructed from these independent predictors and provided a graphical means of estimating the probability of a poor functional outcome based on a cumulative point system. A higher total score indicated a higher predicted clinical risk. The nomogram was then validated using the validation cohort.

Model discrimination was quantified using the area under the receiver operating characteristic curve (AUC). Calibration plots were used to assess the agreement between predicted probabilities and observed outcomes. The clinical utility of the model across a range of threshold probabilities was evaluated using decision curve analysis (DCA) based on net benefit.

## 3 Results

### 3.1 Baseline characteristics

The final analysis included 803 patients, of whom 418 (58.5%) were female, as detailed in Table 1. The study population was categorized into a derivation cohort (*n* = 562) and a validation cohort (*n* = 241). Random allocation resulted in a good balance between the two cohorts, with no significant differences in demographic characteristics, documented risk factors, clinical symptoms, topographic distribution of venous thrombosis, parenchymal lesions, or admission status. Similarly, baseline laboratory biomarkers, including BUN and ALB, were comparably distributed between the two cohorts (Supplementary Table S1).

**Table 1:** Baseline characteristics stratified by functional outcome.

Variable	Good outcome (*n* = 715)	Poor outcome (*n* = 88)	*p*
Demographics			
Age, years, mean ± SD	37.4 ± 13.1	45.1 ± 16.0	<0.001
Female, *n* (%)	418 (58.5)	51 (58.0)	0.927
Possible risk factors, *n* (%)			
Infections	112 (15.7)	24 (27.3)	0.006
Pregnancy/postpartum	105 (14.7)	16 (18.2)	0.387
Clinical symptoms, *n* (%)			
Intracranial hypertension	483 (67.6)	59 (67.0)	0.924
Seizure	161 (22.5)	36 (40.9)	<0.001
Coma	154 (21.5)	60 (68.2)	<0.001
Mental status disturbance	32 (4.5)	10 (11.4)	0.006
^a^Focal neurological deficits	176 (24.6)	37 (42.0)	<0.001
Involved sinuses, *n* (%)			
Transverse sinuses	558 (78.0)	62 (70.5)	0.109
Sigmoid sinuses	477 (66.7)	56 (63.6)	0.564
Superior sagittal sinus	432 (60.4)	52 (59.1)	0.810
Straight sinus	125 (17.5)	37 (42.0)	<0.001
Inferior sagittal sinus	54 (7.6)	8 (9.1)	0.610
Parenchymal lesion, *n* (%)			
Ischemic infarction	147 (20.6)	27 (30.7)	0.030
Intracerebral hemorrhage	109 (15.2)	37 (42.0)	<0.001
Laboratory examinations			
BUN, mg/dL	11.3 ± 4.5	16.3 ± 10.3	<0.001
ALB, g/dL	4.1 ± 0.9	3.7 ± 0.6	<0.001
BAR	2.9 ± 1.3	4.6 ± 3.4	<0.001
Hospital treatment, *n* (%)			
Anticoagulation	669 (93.6)	79 (89.8)	0.184
Endovascular therapies	269 (37.6)	36 (40.9)	0.549

SD, standard deviation; BUN, blood urea nitrogen; ALB, serum albumin; BAR, blood urea nitrogen-to-serum albumin ratio.

a Focal neurological deficits symptoms included hemiplegia and sensory changes.

Clinical characteristics differed between patients with different functional outcomes (Table 2). Patients with poor functional outcomes tended to be older and were more likely to have seizures, coma, and focal neurological deficits at admission. In addition, intracerebral hemorrhage and straight sinus thrombosis were more frequently observed on imaging in the poor-outcome group. The biochemical profiles also differed significantly between the groups, with lower baseline BUN and ALB concentrations observed in patients with good functional outcomes.

**Table 2:** Baseline characteristics stratified by functional outcome in the derivation and validation cohorts.

Variable	Derivation cohort			Validation cohort		
	Good outcome (*n* = 503)	Poor outcome (*n* = 59)	*p*	Good outcome (*n* = 212)	Poor outcome (*n* = 29)	*p*
Demographics						
Age, years, mean ± SD	37.6 ± 13.2	45.7 ± 15.5	<0.001	37.1 ± 13.0	44.0 ± 17.2	0.010
Female, *n* (%)	292 (58.1)	35 (59.3)	0.852	126 (59.4)	16 (55.2)	0.662
Possible risk factors, *n* (%)						
Infections	83 (16.5)	13 (22.0)	0.285	29 (13.7)	11 (37.9)	0.002
Pregnancy/postpartum	73 (14.5)	12 (20.3)	0.237	32 (15.1)	4 (13.8)	0.852
Clinical symptoms, *n* (%)						
Intracranial hypertension	339 (67.4)	41 (69.5)	0.745	144 (67.9)	18 (62.1)	0.529
Seizure	117 (23.3)	23 (39.0)	0.008	44 (20.8)	13 (44.8)	0.004
Coma	108 (21.5)	41 (69.5)	<0.001	46 (21.7)	19 (65.5)	<0.001
Mental status disturbance	24 (4.8)	6 (10.2)	0.081	8 (3.8)	4 (13.8)	0.020
^a^Focal neurological deficits	118 (23.5)	23 (39.0)	0.009	58 (27.4)	14 (48.3)	0.021
Involved sinuses, *n* (%)						
Transverse sinuses	386 (76.7)	40 (67.8)	0.129	172 (81.1)	22 (75.9)	0.502
Sigmoid sinuses	333 (66.2)	35 (59.3)	0.293	144 (67.9)	21 (72.4)	0.626
Superior sagittal sinus	305 (60.6)	32 (54.2)	0.343	127 (59.9)	20 (69.0)	0.348
Straight sinus	81 (16.1)	26 (44.1)	<0.001	44 (20.8)	11 (37.9)	0.039
Inferior sagittal sinus	35 (7.0)	5 (8.5)	0.872	19 (9.0)	3 (10.3)	0.808
Parenchymal lesion, *n* (%)						
Ischemic infarction	99 (19.7)	20 (33.9)	0.011	48 (22.6)	7 (24.1)	0.857
Intracerebral hemorrhage	76 (15.1)	23 (39.0)	<0.001	33 (15.6)	14 (48.3)	<0.001
Laboratory examinations						
BUN, mg/dL	11.3 ± 4.6	16.3 ± 11.8	0.002	11.3 ± 4.3	16.3 ± 6.4	<0.001
ALB, g/dL	4.1 ± 1.0	3.7 ± 0.7	0.008	4.0 ± 0.5	3.7 ± 0.6	0.012
BAR	2.9 ± 1.4	4.7 ± 3.8	<0.001	2.8 ± 1.1	4.6 ± 2.3	<0.001
Hospital treatment, *n* (%)						
Anticoagulation	472 (93.8)	52 (88.1)	0.099	197 (92.9)	27 (93.1)	0.972
Endovascular therapies	194 (38.6)	23 (39.0)	0.951	75 (35.4)	13 (44.8)	0.322

SD, standard deviation; BUN, blood urea nitrogen; ALB, serum albumin; BAR, blood urea nitrogen-to-serum albumin ratio.

a Focal neurological deficits symptoms included hemiplegia and sensory changes.

### 3.2 Clinical predictors of poor functional outcomes in CVST

Multivariable logistic regression identified coma, intracerebral hemorrhage, straight sinus thrombosis, older age, and higher BAR as independent predictors of poor functional outcomes. Straight sinus thrombosis was associated with higher odds of poor functional outcomes, with an adjusted odds ratio (OR) of 4.304 (95% confidence interval [CI], 2.108–8.790). Each one-unit increase in BAR was also associated with higher odds of poor functional outcomes (OR, 1.455; 95% CI, 1.252–1.691). Detailed regression results are presented in Table 3. No substantial multicollinearity was identified among candidate predictors. All candidate predictors showed VIF values ranging from 1.033 to 1.339, indicating no substantial multicollinearity among predictors (Supplementary Table S2).

**Table 3:** Multivariable logistic regression analysis of predictors of poor functional outcome among patients with CVST in the derivation cohort.

Variable	OR	95% CI	*p*
Age	1.041	1.017–1.065	0.001
Coma	8.127	3.894–16.962	<0.001
Seizure	0.829	0.390–1.765	0.627
^a^Focal neurological deficits	1.520	0.775–2.984	0.223
Ischemic Infarction	1.297	0.627–2.683	0.483
Intracerebral hemorrhage	2.397	1.159–4.956	0.018
Straight sinus thrombosis	4.525	2.267–9.031	<0.001
BAR	1.411	1.226–1.625	<0.001

BAR, blood urea nitrogen-to-albumin ratio; OR, odds ratio; CI, confidence interval.

a Focal neurological deficits symptoms included hemiplegia and sensory changes.

### 3.3 Development of a nomogram for poor functional outcomes in CVST

A predictive nomogram was developed (Figure 3) by combining the independent predictors identified in the regression analysis to estimate individual risk. This clinical prediction tool integrates systemic biochemical profiles with neuroimaging features by incorporating age, coma, intracerebral hemorrhage, straight sinus thrombosis, and BAR into a single score to predict poor functional outcomes.

**Figure 3:**
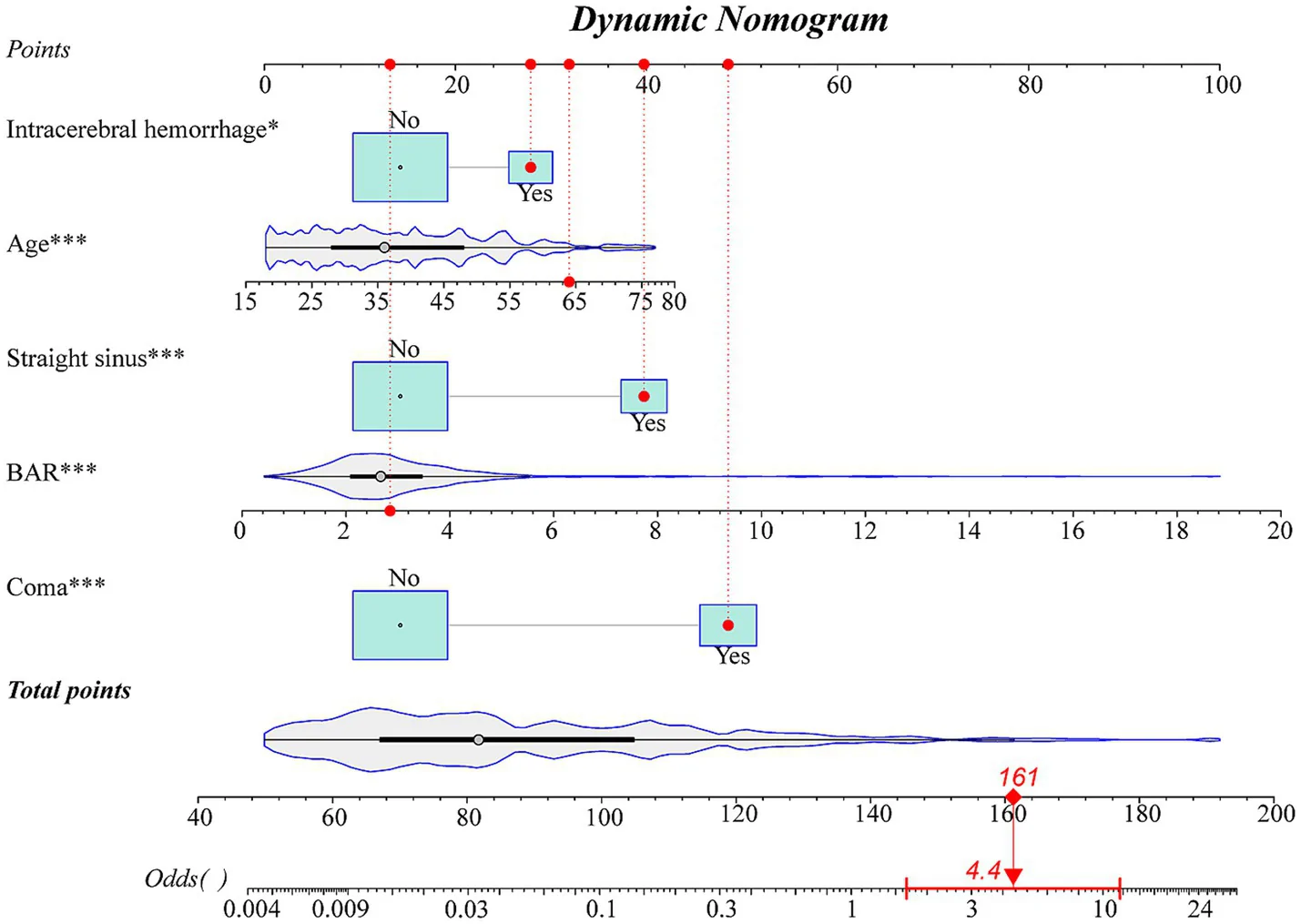
Nomogram for predicting the risk of poor functional outcome in patients with cerebral venous sinus thrombosis. Nomogram for predicting the risk of poor functional outcome in patients with cerebral venous sinus thrombosis. *p < 0.05, ***p < 0.001.

To evaluate the incremental predictive value of BAR, two nested prediction models were developed and compared: a clinical model without BAR and a BAR-incorporated nomogram model. In the derivation cohort, the clinical model achieved an AUC of 0.680, whereas the incorporation of BAR increased the AUC to 0.900 (Figure 4A). Similarly, in the validation cohort, the AUC increased from 0.764 for the clinical model to 0.893 for the BAR-incorporated nomogram model (Figure 4B), demonstrating that incorporating BAR improved discrimination.

**Figure 4:**
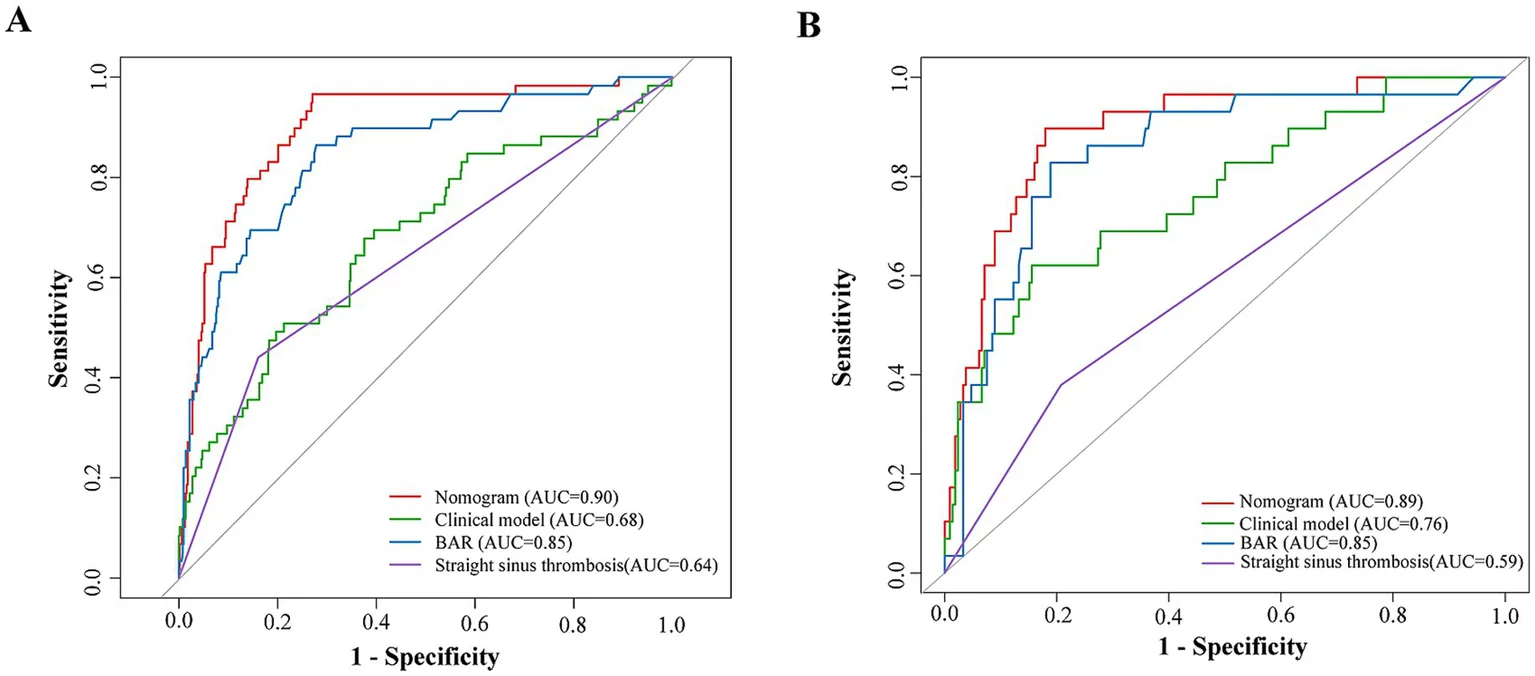
Receiver operating characteristic (ROC) curves comparing the clinical model, nomogram, and individual predictors for predicting poor functional outcome. **(A)** Derivation cohort. **(B)** Validation cohort. The clinical model included age, coma, intracerebral hemorrhage, and straight sinus thrombosis, whereas the nomogram model incorporated BAR in addition to these predictors.

Calibration analysis indicated good agreement between predicted probabilities and observed outcomes for both models. In the derivation cohort, the BAR-incorporated nomogram demonstrated favorable calibration performance, with a Brier score of 0.068 (Figure 5A). Similarly, the validation cohort yielded a Brier score of 0.076, and the calibration curve showed close concordance between predicted risks and actual outcomes across different probability ranges (Figure 5B).

**Figure 5:**
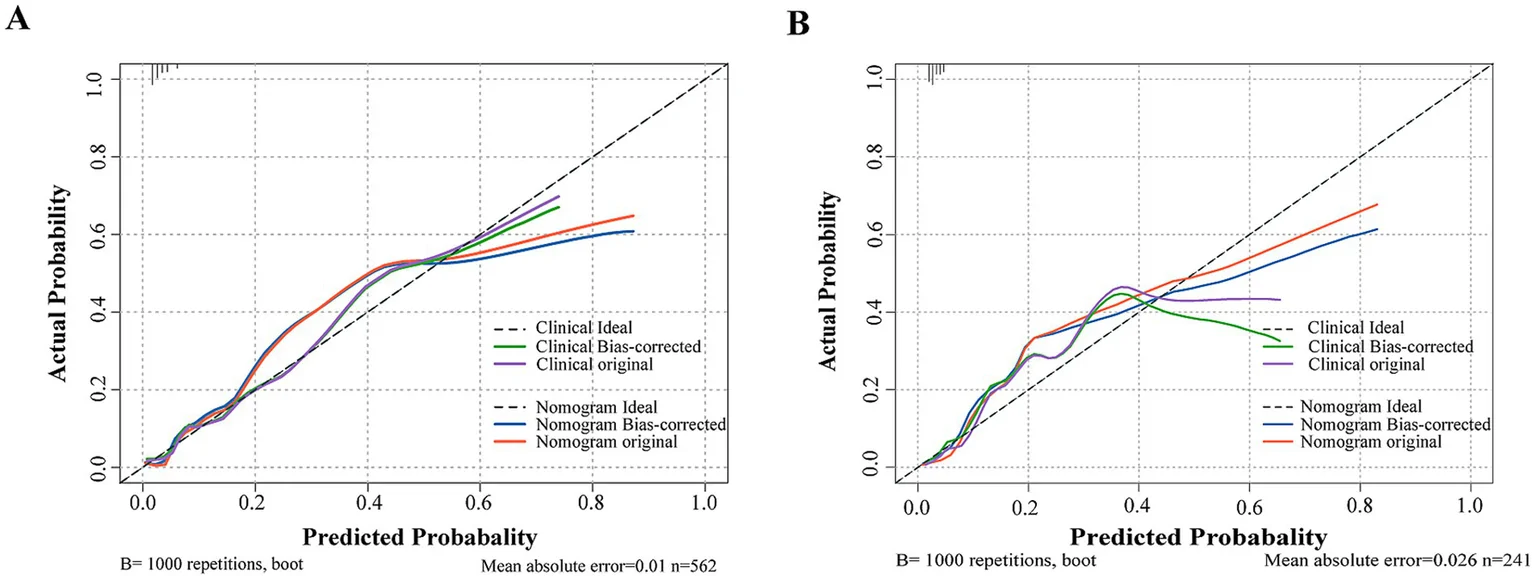
Calibration curves of the clinical model and nomogram in the derivation and validation cohorts. **(A)** Derivation cohort. **(B)** Validation cohort. The clinical model included age, coma, intracerebral hemorrhage, and straight sinus thrombosis, whereas the nomogram model incorporated BAR in addition to these predictors.

DCA demonstrated that the BAR-incorporated nomogram achieved a greater net clinical benefit than the clinical model across a wide range of clinically meaningful threshold probabilities in both the derivation and validation cohorts (Figures 6A,B), suggesting its improved clinical utility for individualized risk stratification.

**Figure 6:**
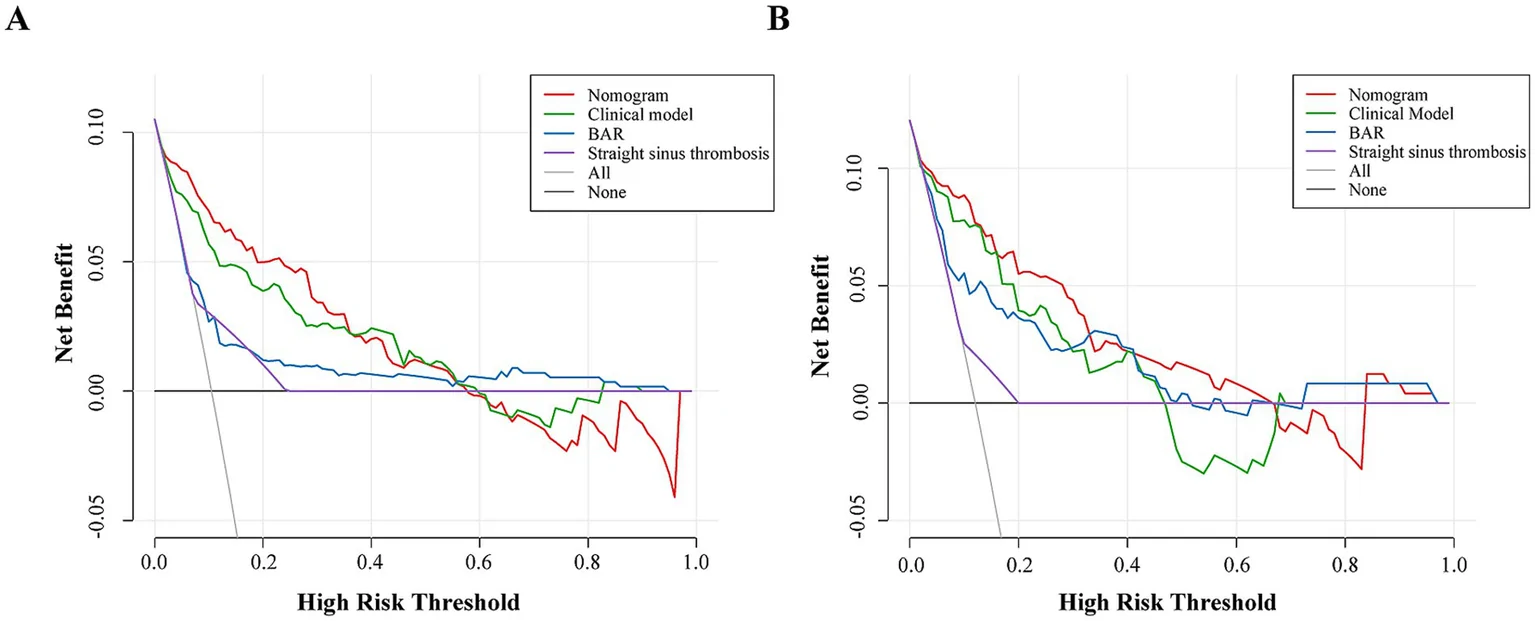
Decision curve analysis (DCA) comparing the clinical model, nomogram, and individual predictors for predicting poor functional outcome. **(A)** Derivation cohort. **(B)** Validation cohort. The clinical model included age, coma, intracerebral hemorrhage, and straight sinus thrombosis, whereas the nomogram model incorporated BAR in addition to these predictors.

To further evaluate the robustness of predictor selection and model performance, additional analyses were performed using the full cohort. In the full-cohort multivariable logistic regression analysis, the same independent predictors identified in the derivation cohort model, including age, coma, intracerebral hemorrhage, straight sinus thrombosis, and BAR, remained independently associated with poor functional outcome (Supplementary Table S3). Furthermore, internal validation of the full-cohort prediction models was performed using 10-fold cross-validation and 1,000 bootstrap resampling iterations. The optimism-corrected performance estimates, including AUC-ROC, Brier score, and calibration slope, demonstrated stable predictive performance after correction for optimism (Supplementary Table S4).

## 4 Discussion

In this observational cohort study, advanced age, coma, intracerebral hemorrhage, radiologically confirmed straight sinus thrombosis, and elevated BAR were independently associated with poor functional outcomes in patients with CVST. A prognostic model was subsequently developed using multivariable logistic regression, integrating these clinical, radiological, and metabolic predictors.

The clinical application of this prognostic nomogram represents a practical addition to traditional, relatively nonspecific clinical grading scales by integrating macrostructural neuroimaging features with systemic metabolic status (31). By incorporating anatomical risk factors, particularly radiologically confirmed straight sinus thrombosis, together with baseline BAR, the model enables objective risk stratification at admission. In clinical practice, it could facilitate the early identification of patients at risk of poor functional outcomes, including those with initially nonspecific presentations in whom metabolic disturbances coexist with a substantial radiological disease burden. This early risk stratification may support closer neurological monitoring, more comprehensive clinical assessment, and individualized management planning. It may also assist clinicians in identifying patients who require greater clinical attention and more intensive monitoring.

The analytical performance of this model is supported by the pathophysiological relevance of its main biomarkers. The adverse clinical profile observed in patients with CVST may be associated with systemic volume depletion, altered hemodynamic status, and increased catabolic stress (2, 32, 33). BUN is a routinely available biochemical parameter that may reflect changes in intravascular volume, renal perfusion, and systemic metabolic stress during acute illness (34, 35). Dehydration has been proposed as a potential contributor to alterations in blood rheology and systemic hemodynamic status. The use of the urea-to-creatinine ratio as a marker of dehydration has been associated with poor functional outcomes. Similar associations between BUN-related indices and early neurological deterioration or poor functional outcomes have also been reported in patients with acute ischemic stroke (36). In the context of CVST, an elevated admission BUN level may reflect volume depletion, acute metabolic stress, or both. Patients with elevated BUN may therefore have greater systemic and neurological disease burdens, depending on the underlying factors contributing to the BUN elevation (37, 38).

In contrast, ALB is a multifunctional plasma protein that maintains colloid oncotic pressure, contributes to systemic antioxidant defense, and reflects inflammatory and nutritional status (39–41). The free thiol group at Cys34 primarily mediates its antioxidant activity by scavenging circulating reactive oxygen and nitrogen species and helping to protect the vascular endothelium from oxidative damage (42). Low ALB levels in acute neurovascular disorders may reflect systemic inflammation, increased vascular permeability, impaired protein metabolism, and depletion of physiological reserves. Disruption of the blood–brain barrier may impair endothelial function and result in fluid leakage, thereby exacerbating cerebral edema and the overall neurological disease burden (43, 44). Accordingly, hypoalbuminemia should not be viewed solely as a marker of malnutrition but may also indicate systemic stress and impaired homeostatic capacity. Previous studies using composite indices that incorporate ALB have reported associations between lower ALB-related measures and poor functional outcomes in patients with CVST. These findings support the potential prognostic relevance of the ALB component of BAR.

As a combination of two biologically complementary laboratory parameters, BAR may better reflect systemic metabolic stress than either BUN or ALB alone. Elevated BUN may indicate volume depletion and increased catabolic activity, whereas decreased ALB may reflect systemic inflammation, nutritional deficiency, and diminished physiological reserve. When combined, these two components may capture clinically relevant changes that could otherwise be overlooked when each biomarker is considered separately. In the present study, a higher baseline BAR was associated with an increased risk of poor functional outcomes in patients with CVST, even after adjustment for established clinical and radiological risk factors. This finding is consistent with accumulating evidence that elevated BAR values are associated with poor functional outcomes in various acute cerebrovascular conditions (23, 26, 38, 45). Given its biological plausibility, routine availability, and low cost, BAR may serve as a useful composite biomarker for early risk stratification in CVST and may complement established clinical and neuroimaging indicators when identifying patients who require closer monitoring and more intensive assessment.

Several methodological limitations should be acknowledged. First, although the nomogram underwent internal validation using a split-sample approach, it has not yet been externally validated in independent cohorts from other institutions. Further multicenter validation is therefore needed to assess its generalizability, calibration, and clinical applicability across different healthcare settings and patient populations. Second, although BAR was calculated based on laboratory measurements obtained within 24 h after admission, the timing of blood sampling relative to treatment initiation was not standardized due to the retrospective nature of the study. Therefore, early therapeutic interventions and fluctuations in hydration status may have affected BUN and albumin levels, potentially introducing measurement bias.

## 5 Conclusion

A practical prognostic nomogram integrating baseline BAR with key clinical and radiological factors was developed to identify patients with CVST at increased risk of poor functional outcomes.

## Data Availability

The datasets generated and analyzed during the current study are not publicly available because they contain sensitive patient information and are subject to institutional and ethical restrictions. Requests to access de-identified data may be directed to the corresponding author (BS, fccsongb@zzu.edu.cn) and will be considered subject to approval by the relevant ethics committee and institution.
