# Supplementary material for: Prognostic model integrating the blood urea nitrogen-to-serum albumin ratio and radiological features for poor functional outcome in cerebral venous sinus thrombosis
Source: Front Neurol. 2026 Oct 21;17:1953972. doi: 10.3389/fneur.2026.1953972 (PMC13730841; doi:10.3389/fneur.2026.1953972)
Supplement: Supplementary file 1 [file Table_1.docx]

**Supplementary Table S1.** Baseline characteristics of the derivation and validation cohorts

|  | Derivation cohort (n=562) | Validation cohort (n=241) | *P* |
| --- | --- | --- | --- |
| Demographics |  |  |  |
| Age, years, mean ± SD | 38.4±13.7 | 37.9±13.7 | 0.651 |
| Female, n (%) | 327(58.2) | 142(58.9) | 0.846 |
| Possible Risk factors, n (%) |  |  |  |
| Infections | 96(17.1) | 40(16.6) | 0.867 |
| Pregnancy/postpartum | 85(15.1) | 36(14.9) | 0.946 |
| Clinical symptoms, n (%) |  |  |  |
| Intracranial hypertension | 380(67.6) | 162(67.2) | 0.913 |
| Seizure | 140 (24.9) | 57(23.7) | 0.704 |
| Coma | 149(26.5) | 65(27.0) | 0.893 |
| Mental status disturbance | 30(5.3) | 12(5.0) | 0.834 |
| ^a^Focal neurological deficits | 141(25.1) | 72(29.9) | 0.159 |
| Involved sinuses, n (%) |  |  |  |
| Transverse sinuses | 426(75.8) | 194(80.5) | 0.146 |
| Sigmoid sinuses | 368(65.5) | 165(68.5) | 0.412 |
| Superior sagittal sinus | 337(60.0) | 147(61.0) | 0.784 |
| Straight sinus | 107(19.0) | 55(22.8) | 0.221 |
| Inferior sagittal sinus | 40(7.1) | 22(9.1) | 0.328 |
| Parenchymal lesion, n (%) |  |  |  |
| Ischemic Infarction | 119(21.2) | 55(22.8) | 0.604 |
| Intracerebral hemorrhage | 99(17.6) | 47(19.5) | 0.525 |
| Laboratory Examinations |  |  |  |
| BUN, mg/dL | 11.9±6.0 | 11.9±5.0 | 0.995 |
| ALB, g/dL | 4.0±1.0 | 4.0±0.6 | 0.259 |
| BAR | 3.0±1.9 | 3.1±1.4 | 0.944 |
| Hospital treatment, n (%) |  |  |  |
| Anticoagulation | 524(93.2) | 224(92.9) | 0.881 |
| Endovascular Therapies | 217(38.6) | 88(36.5) | 0.575 |

**Abbreviations:** SD, standard deviation; BUN, blood urea nitrogen; ALB, serum albumin; BAR, blood urea nitrogen-to-albumin ratio; ^a^Focal neurological deficits symptoms included hemiplegia and sensory changes
