# Supplementary material for: Prognostic model integrating the blood urea nitrogen-to-serum albumin ratio and radiological features for poor functional outcome in cerebral venous sinus thrombosis
Source: Front Neurol. 2026 Oct 21;17:1953972. doi: 10.3389/fneur.2026.1953972 (PMC13730841; doi:10.3389/fneur.2026.1953972)
Supplement: Supplementary file 2 [file Table_2.docx]

**Supplementary Table S2.** Assessment of multicollinearity among candidate predictors

| Variable | Tolerance | VIF |
| --- | --- | --- |
| Age | 0.947 | 1.056 |
| Coma | 0.749 | 1.335 |
| Seizure | 0.747 | 1.339 |
| ^a^Focal neurological deficits | 0.920 | 1.087 |
| Ischemic Infarction | 0.953 | 1.049 |
| Intracerebral hemorrhage | 0.894 | 1.119 |
| Straight sinus thrombosis | 0.968 | 1.033 |
| BAR | 0.967 | 1.034 |

**Abbreviations:** BAR, blood urea nitrogen-to-albumin ratio; VIF, variance inflation factor; ^a^Focal neurological deficits symptoms included hemiplegia and sensory changes
