# Supplementary material for: Prognostic model integrating the blood urea nitrogen-to-serum albumin ratio and radiological features for poor functional outcome in cerebral venous sinus thrombosis
Source: Front Neurol. 2026 Oct 21;17:1953972. doi: 10.3389/fneur.2026.1953972 (PMC13730841; doi:10.3389/fneur.2026.1953972)
Supplement: Supplementary file 3 [file Table_3.docx]

**Supplementary Table S3.** Full-cohort multivariable logistic regression analysis of predictors of poor functional outcome in patients with CVST

| Variable | OR | 95% CI | *P* |
| --- | --- | --- | --- |
| Age | 1.037 | 1.018-1.057 | <0.001 |
| Coma | 6.443 | 3.573-11.617 | <0.001 |
| Seizure | 0.956 | 0.518-1.766 | 0.886 |
| ^a^Focal neurological deficits | 1.580 | 0.915-2.73 | 0.101 |
| Ischemic Infarction | 1.298 | 0.716-2.354 | 0.391 |
| Intracerebral hemorrhage | 2.857 | 1.592-5.127 | <0.001 |
| Straight sinus thrombosis | 3.638 | 2.075-6.378 | <0.001 |
| BAR | 1.460 | 1.285-1.658 | <0.001 |

**Abbreviations:** BAR, blood urea nitrogen-to-albumin ratio; OR, odds ratio; CI, confidence interval; ^a^Focal neurological deficits symptoms included hemiplegia and sensory changes
