# Supplementary material for: Prognostic model integrating the blood urea nitrogen-to-serum albumin ratio and radiological features for poor functional outcome in cerebral venous sinus thrombosis
Source: Front Neurol. 2026 Oct 21;17:1953972. doi: 10.3389/fneur.2026.1953972 (PMC13730841; doi:10.3389/fneur.2026.1953972)
Supplement: Supplementary file 4 [file Table_4.docx]

**Supplementary Table S4.** Internal validation performance of the full-cohort prediction models using 10-fold cross-validation and bootstrap resampling

| Validation method | Performance metric | Clinical model | Nomogram |
| --- | --- | --- | --- |
| 10-fold cross-validation | Mean AUC | 0.841 | 0.885 |
|  | Mean Brier score | 0.080 | 0.075 |
| Bootstrap validation  (1,000 resamples) | Apparent AUC | 0.846 | 0.891 |
|  | Optimism-corrected AUC | 0.841 | 0.892 |
|  | Apparent Brier score | 0.078 | 0.072 |
|  | Optimism-corrected Brier score | 0.079 | 0.074 |
|  | Apparent calibration slope | 1.000 | 1.000 |
|  | Optimism-corrected calibration slope | 0.974 | 0.959 |

**Abbreviations:** AUC, area under the receiver operating characteristic curve; The clinical model included age, coma, intracerebral hemorrhage, and straight sinus thrombosis, whereas the nomogram model incorporated BAR in addition to these predictors.
